# Supplementary material for: The Effectiveness of a Four-Week Digital Physiotherapy Intervention to Improve Functional Capacity and Adherence to Intervention in Patients with Long COVID-19
Source: Int J Environ Res Public Health. 2022 Aug 3;19(15):9566. doi: 10.3390/ijerph19159566 (PMC9367987; doi:10.3390/ijerph19159566)
Supplement: Supplementary file 1 [file ijerph-19-09566-s001.zip › ijerph-1823030-supplementary.pdf]

File S1

TIDieR Template for Intervention Description and Replication

The TIDieR (Template for Intervention Description and Replication) Checklist*: ✓

Information to include when describing an intervention and the location of the information

Item number	Item<br>The Effectiviness of a four-week Digital physiotherapya intervention to improve functional capacitiy and adherence in patients with Long COVID-19	Where located ** Primary paper (page or appendix number)	Other † (details)
	**BRIEF NAME**		
1.	Provide the name or a phrase that describes the intervention.	1	
	**WHY**		
2.	Describe any rationale, theory, or goal of the elements essential to the intervention.	2-5	
	**WHAT**		
3.	Materials: Describe any physical or informational materials used in the intervention, including those provided to participants or used in intervention delivery or in training of intervention providers. Provide information on where the materials can be accessed (e.g. online appendix, URL).	6-8	
4.	Procedures: Describe each of the procedures, activities, and/or processes used in the intervention, including any enabling or support activities.	6-8	
	**WHO PROVIDED**		
5.	For each category of intervention provider (e.g. psychologist, nursing assistant), describe their expertise, background and any specific training given.	5	
	**HOW**		
6.	Describe the modes of delivery (e.g. face-to-face or by some other mechanism, such as internet or telephone) of the intervention and whether it was provided individually or in a group.	6-8	
	**WHERE**		
7.	Describe the type(s) of location(s) where the intervention occurred, including any necessary infrastructure or relevant features.	6-7	
	**WHEN and HOW MUCH**		
8.	Describe the number of times the intervention was delivered and over what period of time including the number of sessions, their schedule, and their duration, intensity or dose.	6-7	
	**TAILORING**		
9.	If the intervention was planned to be personalised, titrated or adapted, then describe what, why, when, and how.	6	
	**MODIFICATIONS**		
10.ǂ	If the intervention was modified during the course of the study, describe the changes (what, why, when, and how).	NA	
	**HOW WELL**		
11.	Planned: If intervention adherence or fidelity was assessed, describe how and by whom, and if any strategies were used to maintain or improve fidelity, describe them.	9	
12.ǂ	Actual: If intervention adherence or fidelity was assessed, describe the extent to which the intervention was delivered as planned.	13	

** **Authors**—use N/A if an item is not applicable for the intervention being described.
Reviewers—use '?' if information about the element is not reported/not sufficiently reported.

† If the information is not provided in the primary paper, give details of where this information is available. This may include locations such as a published protocol
or other published papers (provide citation details) or a website (provide the URL).

ǂ If completing the TIDieR checklist for a protocol, these items are not relevant to the protocol and cannot be described until the study is complete.

* We strongly recommend using this checklist in conjunction with the TIDieR guide (see BMJ 2014;348:g1687) which contains an explanation and elaboration for each item.

File S2

Informed Consent—Written Consent of The Patient

COVID-19 can cause important sequelae in the patient after overcoming the virus, such as: fatigue, joint pain, headaches, sleep disorders and respiratory problems, among other symptoms. Physiotherapy has been shown to play a fundamental role in the recovery of functions and quality of life and its effectiveness in patients who have been infected by this virus is under continuous study.

Digital physiotherapy practice is a term used to describe the provision of rehabilitation services at a distance, using communication technologies, such as mobiles, tablets and computers, which can better meet the needs of the patient, in terms of ease of access and elimination of travel and in the case of COVID-19 limiting its potential transmission by avoiding direct person-to-person contact. Digital physiotherapy practice has increased significantly during periods of confinement and is presented as an intervention opportunity for patients.

WHAT DOES THIS RESEARCH PROJECT CONSIST OF?

We want to test the effectiveness of digital physiotherapy practice in Long COVID-19 patients for the improvement of their functional capacities. We also investigated the level of adherence to treatment.

Patients participating in the research are assessed by the research team to identify their particular health needs. For 4 weeks, they will receive personalised physiotherapy sessions in digital format using technological tools via their mobile phone, computer or tablet. The researchers will inform them of the recommendations to be followed during the evolution of the sessions and the treatment to be carried out after the treatment period.

The research project has been approved by the Research Ethics Committee of Andalusia. The interventions to be carried out have the maximum guarantees of good professional practice, safety and data protection. Your case will be closely monitored by the researchers, who include professionals from the Universities of Malaga and Granada with more than 25 years of professional and research experience.

What do You Need to Participate?

- Be of legal age
- Diagnosis: Long COVID-19
- Have a mobile phone, tablet or computer at home and an internet connection.
- Complete and sign the informed consent document attached below.

Informed Consent—Written Consent of The Patient

I (Name and surname): ....

1. I declare that I have read the Patient Information Sheet that accompanies this consent.

2. I was able to ask questions about the study. All questions were answered to my satisfaction.

3. I have spoken to the reporting health professional: ………………………………………………

4. I understand that my participation is voluntary and I am free to participate or not in the study.

5. I have been informed that all data obtained in this study will be confidential and will be treated in accordance with the provisions of Organic Law 3/2018 of 5 December.

6. I understand that I can withdraw from the study:

- Whenever you want
- Without having to explain
- Without impacting on my medical care

I freely give my agreement to participate in the project entitled " Digital physiotherapy practice in Patients with Long COVID-19 ".

I DO

I DO NOT GIVE

Signature of the patient Signature of the informing health professional

First and last name: ....First and last name: ………………………………

Date: ....

Contact telephone: ....
